# Cutaneous Melanoma—A Long Road from Experimental Models to Clinical Outcome: A Review

**DOI:** 10.3390/ijms19061566

**Published:** 2018-05-24

**Authors:** Dorina Coricovac, Cristina Dehelean, Elena-Alina Moaca, Iulia Pinzaru, Tiberiu Bratu, Dan Navolan, Ovidiu Boruga

**Affiliations:** 1 Department of Toxicology, Faculty of Pharmacy, “Victor Babeş” University of Medicine and Pharmacy, 300041 Timişoara, Romania; dorinacoricovac@umft.ro (D.C.); cadehelean@umft.ro (C.D.); alina.moaca@umft.ro (E.-A.M.); iuliapinzaru@umft.ro (I.P.); 2 Faculty of Medicine, “Victor Babeş” University of Medicine and Pharmacy, 300041 Timişoara, Romania; drbratu@brol.ro (T.B.); ovidiuboruga@yahoo.com (O.B.); 3 Department of Obstetrics-Gynecology and Neonatology, Faculty of Medicine, “Victor Babes” University of Medicine and Pharmacy, 300041 Timișoara, Romania

**Keywords:** cutaneous melanoma, incidence, etiology, animal models, genetic profile

## Abstract

Cutaneous melanoma is a complex disorder characterized by an elevated degree of heterogeneity, features that place it among the most aggressive types of cancer. Although significant progress was recorded in both the understanding of melanoma biology and genetics, and in therapeutic approaches, this malignancy still represents a major problem worldwide due to its high incidence and the lack of a curative treatment for advanced stages. This review offers a survey of the most recent information available regarding the melanoma epidemiology, etiology, and genetic profile. Also discussed was the topic of cutaneous melanoma murine models outlining the role of these models in understanding the molecular pathways involved in melanoma initiation, progression, and metastasis.

## 1. Introduction

Melanoma, the malignancy that is referred to as “the cancer that rises with the Sun” [1], originates from melanocytes that switched to cancerous cells as a consequence of aberrant changes at molecular and biochemical levels [2]. Albeit melanoma adds up the smallest number of skin cancer cases (<10%) [3], its aggressiveness and its high mortality rate make it the deadliest type of skin cancer. In the last four to five decades, a constant increase of melanoma incidence was recorded, placing this malignant disorder on the 19th position worldwide among the most common cancer types [1], whereas when referring to individual countries, the highest incidence was recorded in Australia (fourth place) [4], New Zealand, United States (Northern Region), and European countries (Northern and Western regions), and a lower incidence in South-Eastern Asia and South-Central Asia [5,6].

Melanoma is characterized by an extensive degree of heterogeneity in terms of clinical, dermatological, and histopathological presentation [4], genomic profile [7,8], and risk factors (skin type, exposure to sun radiation, number of nevi, age, gender, immune status, family history or former removed melanomas) [9], which awards this disorder as a significant public health issue and an important matter of concern for the scientists in the field of cancer. The impressive number of results achieved at a simple search in PubMed database for “melanoma” (over 8800 papers were published since 1 January 2017 up to present, whereas all-time publications exceeded 119,000 articles) witnesses the magnitude of this concern.

This paper offers a concise overview of the latest data concerning the epidemiology, etiology, genetic profile, and the state of the art of murine models of cutaneous melanoma, highlighting the contribution of these models in understanding the molecular mechanisms of melanoma. 

## 2. Epidemiology and Etiology of Cutaneous Melanoma

Early stage diagnosed melanoma that was surgically removed is considered a curable pathology with a good prognosis, whereas the treatment options for advanced/metastatic melanoma remain poor [4]. On this basis, many efforts were done worldwide in the last decades for the prevention and early diagnosis of melanoma, as follows: campaigns to reduce hazardous sun exposure, sun protection policies [10,11], national primary prevention efforts in the 1990s and the SunSmart Campaign—2003 (in United Kingdom), primary prevention messages communicated by the national cancer societies and radiation safety agencies (in Norway and Sweden), mass-media campaigns in the early 1980s (in Australia and New Zealand) [11], collection of the melanoma data in population-based European cancer registries [12], Euromelanoma campaign and EUROCARE 5 [13], and web platforms—The Virtual Melanoma Cell—used to enable the disease-specific mining of high-throughput data [14]. Moreover, in order to improve the early detection of melanoma (which is associated with a reduced mortality), a novel approach was suggested: the use of smartphones apps and the store-and-forward teledermatology via a smartphone [15]. Another tailored prevention form consisted of the development of a telephone communication protocol for disclosing melanoma genomic risk information to the asymptomatic general population after a sample of their saliva was tested for melanoma [16].

Given the estimates of GLOBOCAN and WHO concerning the future burden of melanoma, a high interest was assigned to the trends of melanoma incidence and mortality rates taking into consideration parameters as age, gender, geographical localization, ultraviolet (UV) exposure, and tumor thickness and invasiveness, which are topics described in numerous epidemiological studies and meta-analyses [11,12,13,17,18,19]. In a study that included the data collected from 11 countries (Bosnia and Herzegovina, Bulgaria, Croatia, Cyprus, Czech Republic, Malta, Romania, Serbia, Slovakia, Slovenia, and Turkey) from South-Eastern Europe, it was analyzed the age-specific incidence and mortality trends of melanoma and were drawn the following conclusions: (i) the incidence rates augmented uniformly over the 2000–2010 period, but at a lower extent as compared to North-Western Europe; (ii) the incidence was higher in men as compared to women at middle (50–69 years) and older (70+ years) ages in most countries; and (iii) the mortality trends were less favorable than in North-Western Europe [13]. Whiteman and collaborators analyzed recent trends and estimated future melanoma incidence (a projection until 2031) in six populations with European heritage in terms of UV exposure patterns and different approaches to melanoma control and found an increasing trend of melanoma incidence in United States white population, in United Kingdom, Swedish, and Norwegian populations, a steady incidence in New Zealand, and a decreasing one in Australia [11]. A very recent article investigated melanoma incidence and mortality in 13 European countries (during 1995–2012 period) by country, age, sex, and Breslow thickness, and the results showed that the incidence of invasive melanoma continues to increase, mainly due to thin lesions, with the greatest increase in the Netherlands, the mortality trend being also an ascending one in most countries [12].

In spite of all the preventive measures, informative campaigns for early detection of melanoma and novel targeted therapies, the incidence of melanoma still keeps an ascending trend worldwide, and the causal factors of melanomagenesis remain under debate.

Cutaneous melanoma, which arises from epidermal melanocytes, is described as a disorder of people that present a fair-skinned phenotype, a family history of melanoma, and erratic genetic risk factors [20]. The melanocytes genitors are considered to be the neural crest progenitor cells that differentiate during embryonic development via “dorsolateral” pathway into melanoblasts which migrate to dermis where the differentiation process results in melanocytes that will further migrate to the epidermis [2]. Melanocytes are responsible for pigment production—melanins (black eumelanin and pheomelanin) that play key roles in offering protection against DNA damage induced by UV radiation [21], thus recent studies assert the duality of these molecules (mainly for pheomelanin) in melanomagenesis, the photodamage exceeding the photoprotection [22,23,24]. The synthesis of melanin (melanogenesis) is regulated by a multitude of agents (including hormones) that interact via pathways triggered by receptor-dependent and independent mechanisms, in hormonal, auto-, para-, or intracrine manner (the positive regulators of melanogenesis: MC1 receptor, melanocortins, ACTH—adrenocorticotropic hormone, l-tyrosine and l-dihydroxyphenylalanine—l-DOPA) [25,26]. Moreover, it was proven that melanogenesis influences melanoma behavior [27] and its response to therapy [28,29], and it also exerts an immunosuppressive effect [30,31]. The regulation of melanin transport following ultraviolet exposure together with the control of melanocytes growth and melanin synthesis is accomplished by keratinocytes. Once the keratinocytes lose control over melanocytes, the latter start to grow in an uncontrolled manner and acquire the ability to migrate out of skin, processes that indicate the development of melanoma [32].

The role of ultraviolet (UV) radiation in the melanoma occurrence is not fully elucidated and seems to be complex (since only a reduced number of UV signature mutations were detected in melanoma patients as compared to the ones diagnosed with non-melanoma skin cancers), but it was stated that intermittent UV exposure is linked to melanoma development [18]. In the case of cutaneous melanoma development, the risk is higher after a number of episodes of intense UV exposure (sunburn) during childhood (before the age of 10), as compared to the other skin malignancies when the risk of development is correlated to lifetime exposure to UV radiation [33]. Genomic and next generation sequencing studies attested the role of UV radiation as the main mutagen in cutaneous melanoma. Moreover, it was demonstrated that in invasive melanoma the number of UV-induced mutations is higher than the number of nevi (being included here also the matched precursor nevi), and the consequences are increased somatic mutation burdens [33,34]. 

Other genetic and phenotype factors were discussed to be involved in melanoma development, like: gender, age, skin type, number of nevi (>50 moles—high risk), family history, immune status, etc. [9,21]. There were also reported gender disparities concerning the incidence of melanoma in women versus men (with an increasing trend in men), referring to physiologic differences in skin structure, baseline differences in immune systems, the influence of sex hormone levels, and estrogen receptor expression [18,35].

Cutaneous melanoma was described as one of the most immunogenic cancers with heterogeneous histological and clinical features, and a significant number of mutations, which explains the low rate of tumor regression, multi-drug resistance to targeted therapies, and reduced survival rate [21,36]. The aggressiveness of melanoma could be explained by the ability of melanoma cells to escape apoptosis by overexpressing the apoptosis-inhibitory genes (as survivin and other inhibitory apoptosis proteins—IAPs) or by reducing the apoptosis-stimulatory genes expressions what leads to apoptosis failure and an augmented risk of metastasis [37]. In order to have a clear picture of melanoma initiation, progression, and metastasis, it is imperative to have the basic knowledge about these processes stages and the underlying mechanisms involved.

The development of melanoma occurs in a stepwise manner from benign nevus to invasive melanoma, a model proposed by Wallace Clark and collaborators that includes six steps very well defined in clinical and histopathological terms [33,38]. The starting point is a benign nevus composed of a clonal population of melanocytes that were aberrantly transformed into a hyperplastic lesion which will not advance due to cellular senescence. The following step is represented by the radial growth phase (RGP) that consists of nevus transformation into a dysplastic lesion that will evolve to a superficial spreading stage, confined to epidermis with low invasive potential. In the final step, also known as the vertical growth phase, the cells from the radial growth phase acquire the capacity to invade the dermis and metastasize [39,40]. Another aspect that should be kept in mind is the tumor vascularization—the supplier of the nutrients which, until the tumor reaches the size of 2–3 mm, occurs naturally by passive diffusion. After the tumor becomes bigger than 2–3 mm, the angiogenesis process, the new blood vessel formation, is initiated in order to sustain the needs of the melanoma cells. Once the tumor is amply vascularized, the mass of the tumor augments [36]. Some studies assert the idea that melanoma development is accompanied by an epithelial-to-mesenchymal (EMT) switch characterized by the melanocytes loss of E-cadherin expression and acquisition of some mesenchymal markers as SNAIL (transcription factor of zinc-finger family), SLUG (transcriptional repressor of E-cadherin), TWIST (Twist-related protein 1), and ZEB1 (Zinc finger E-box binding homeobox 1 transcription factor) [32,41].

A number of histological subtypes was described for cutaneous melanoma, like: superficial spreading melanoma, nodular melanoma, polypoid melanoma, acral lentiginous melanoma, lentigo maligna melanoma, and some uncommon forms: desmoplastic melanoma, nevoid melanoma, amelanotic melanoma, and verrucous melanoma [4,7,21]. The variability of melanoma in both clinical presentation and dermatoscopical features, and sometimes the lack of these specific features, becomes a challenge in establishing the diagnosis and even brought it the name of “the great imitator” [4].

When it comes to predict the outcome of primary tumors, it is applied the 2009 American Joint Committee on Cancer (AJCC) Melanoma Staging and Classification System that evaluates: tumor thickness, ulceration, mitotic figures, and microscopic satellites [42,43]. The number of mitoses (established by histopathological measurements) is an important prognostic factor for thin melanomas (Breslow thickness < 0.75 mm) and was included in the 7th classification of AJCC. The dermoscopic features like black color and peripheral streaks are positively correlated with thin melanomas with mitoses, while brown color and atypical pigment network are associated with a less aggressive phenotype [44]. According to the National Comprehensive Cancer Network guidelines, the patients who have a Breslow index of 0.76–1.0 mm with no ulceration or mitotic rate are not subjected for sentinel lymph node biopsy. Based on the 2009 AJCC staging system, mitotic rate higher than or equal to 1 mitosis/mm^2^ was correlated with poor disease-specific survival, especially in patients with a melanoma thickness less than or equal to 1.0 mm thick [45,46].

## 3. Cutaneous Melanoma Genetic Profile

One of the features that places melanoma in the top of the most aggressive type of cancer is represented by its heterogeneous nature. The intratumor and intertumor heterogeneity in melanoma were explained very comprehensively in an excellent recent review [45]. According to Grzywa et al, the intratumor heterogeneity is characterized by genomic instability (having as result the acquisition of common mutations—which occur early in tumor evolution and are found in all regions, of shared/branch mutations—that occur later and were detected only in some regions, and of private mutations—that occur in tumor progression phase, present in a single compartment), genomic and epigenomic alterations (having as consequence heterogeneous genes expression), and epigenetic dysregulation [45]. The complexity of melanoma is also conferred by the myriad of signaling pathways involved in melanoma development, pathways that coincide with the ones required for melanocytes development, like: Notch, Wnt, endothelins, SOX (sex-determining region Y–like–SRY high-mobility group—HMG box) proteins, mitogen-activated protein kinase (MAPK) signaling pathway, phosphatidylinositol-3-kinase (PI3K) pathway, G-protein-coupled receptor (GPCR) family, and epithelial-to-mesenchymal transition [40,46].

A considerable progress was registered in the genomic field of melanoma in recent years, a major role being played by the novel techniques like: next-generation sequencing and large-scale expression analyses of tumors which offer a landscape of the mutations existent in melanoma, the mutation rate in melanoma exceeding all the other cancers rates (the number of mutations per Mb ranged from 0.1 to 100 with an average value of 16.8 mutations/Mb according to The Cancer Genome Atlas (TCGA) data) [45,47].

The molecular pathways and the genes involved in melanomagenesis were discussed extensively in retrospective [2,39,40,48,49] and recent studies [26,45,50,51,52,53], this being the reason why the present review will discuss only the genes involved in the development of cutaneous melanoma in a concise manner.

The multitude mutations discovered to be engaged in melanoma increases the difficulty in identifying which are the “driver” (causative) mutations and the “passenger” (bystander) mutations [47,53]. A very detailed description of the genes known to be altered in melanoma, together with their impact in melanomagenesis and their potential to become targets for therapy, was compiled by Shtivelman and coauthors [47]. Based on the results of a whole-genome sequencing analysis, the genes susceptible to mutations in cutaneous melanoma are: *BRAF*, cyclin-dependent kinase N2A (*CDKN2A*), *NRAS*, and *TP53* [53].

*BRAF* is a serine-threonine kinase involved in cell proliferation that triggers MAPK (mitogen-activated protein kinase) signaling pathway after its activation by *RAS* family of proteins. MAPK pathway controls important cellular processes, as: cell cycle progression, differentiation, and upregulation of transcription, and the existence of *BRAF* mutations will determine impairment of these processes, the end-point being oncogenesis [8,50,52]. *BRAF* mutations are very common in cutaneous melanoma and trigger MAPK pathway activation (60% of the cutaneous melanomas exhibit MAPK activating mutations), whereas in other types of melanoma, such as acral, mucosal, conjunctival, and uveal, its incidence is quite low [50,53].

Valine-to-glutamic acid substitution at codon 600 (*BRAF* (*V600E*)) is the most prevalent mutation in melanoma (detected in approximately 50% of melanomas) and might be the repercussion of a secondary effect of UV damage, like a nonclassic DNA mutation induced by UV radiation or the synthesis of reactive oxygen species [7,53]. This mutation was reported in melanomas and melanocytic nevi, leading to activation of RAS/RAF/MEK/ERK pathway, a key player in the initiation of melanocytic tumors [7,8]. A novel *BRAF* mutation (an aminoacidic insertion in codon 599) was identified in a melanoma patient in the P-loop activating site, mutation that was not discovered before in melanoma, but was detected rarely in Papillary Thyroid Carcinoma and Anaplastic Thyroid Carcinoma, which highlights the heterogeneity of this disease [54].

*NRAS* mutations represent the second most frequent cause of altered signaling via MAPK pathway. This type of mutations was identified in 15–30% of melanomas and were found at codon 12, 13, or 61. Of note, *NRAS* and *BRAF* mutations are mutually exclusive, the presence of co-mutations was rarely observed, and in order to trigger malignant transformation, additional mutations are required, such as loss of tumor suppressors p16^INK4A^ (Cyclin-dependent kinase inhibitor 2A) or PTEN (phosphatase and tensin homolog protein) [51,52]. The consequences of activated *BRAF* or *NRAS* mutations consist of aberrant cell growth, followed by premature growth arrest via oncogene-induced senescence, the resulted lesions remain benign and do not switch to malignancy in the absence of other mutations [55].

NF1 protein, also known as neurofibromin 1, is considered a “driver” mutation in a subset of melanoma. NF1 mutations were associated with initiation of melanoma and are prevalent in chronically sun-exposed skin. In addition, regulates negatively RAS family leading to RAF inhibitor resistance. To note, NF1 mutations or suppression might appear in parallel to *BRAF* mutations [51,52,53,54].

The cyclin-dependent kinase inhibitor 2A gene (*CDKN2A*) is the familial melanoma locus (located on the short arm of chromosome 9) that controls two tumor suppressor proteins (p14-ARF and p16-INK4A) with major roles in cell proliferation and senescence [7,51]. *CDKN2A* mutations were reported in approximatively 15% of familial melanomas, the somatic defects happened as a result of an impaired or loss of function (mutations, homozygous deletions or DNA methylation-induced epigenetic silencing) and are correlated with an invasive potential. These mutations are prevalent in melanomas (in 90%) and in dysplastic nevi (10%), and are not expressed in common melanocytic nevi [2,51,53,55].

## 4. Murine Models of Melanoma

The information acquired to offer a complete picture of melanoma etiology and progression was relied on melanoma models. According to Herlyn and Fukunaga-Kalabis, in 2010, it was estimated a number of 5000 cell lines developed by different laboratories, and over 200 of these melanoma cell lines were characterized in terms of genetic aberrations, gene expressions patterns, and biological properties (in vitro invasion ability, tumor development, and metastasis in immunodeficient mice) [56]. Despite the considerable amount of data that was provided by the use of melanoma cell lines, these models present several limitations, such as: a different behavior of cells in culture conditions as the ones in a patient’s body, the interactions with the tumor environment cannot be recreated in vitro, and each melanoma cell behaves as a stem cell due to its capacity of self-renewal and to develop tumors [56]. Other approach designed to overcome the limitations of the in vitro melanoma models and of the xenograft mouse models (differences between human and murine skin architecture, disparities in histopathological features, incapacity to recreate the initial events involved in the early invasion through the basement membrane) was a fully humanized 3D skin equivalent to early melanoma invasion model [57]. The number of relevant animal melanoma models available in the literature in the last decades have hampered the paucity concerning the cellular processes involved in melanoma initiation, progression, and metastasis, and also the key genes related to tumorigenesis [20]. An ideal model of melanoma would be considered the model that recreates human disease, having an UV-based etiology, the histopathological features of cutaneous melanoma and its molecular genetic fingerprint, and can be subjected to genetic and immunologic manipulation [58]. Although there were obtained multiple animal models of melanoma using large animals (horses, dogs (reviewed in [20]), Sinclair swine [21,59]) and small animals (zebrafish [60,61], opossum—Monodelphis domestica—[58], gerbils and hamsters [62,63,64], and mice [2,20,21,39,50,58,65]), the ideal melanoma model that accomplishes all the requirements mentioned above was not developed yet.

Experimental animal models also proved to be reliable sources of data concerning (i) the screening for novel antimelanoma agents: temozolomide (B16F10 metastatic melanoma model) [66,67], thymoquinone (B16F10 intracerebral melanoma model using C57BL/6J mice as host) [68], oncolytic herpes simplex virus HF10, and dacarbazine combined therapy (DBA/2 mice subcutaneously inoculated with clone M3 mouse melanoma cells) [69], gliotoxin (a xenograft mouse model using athymic mice) [70], recombinant methioninase [71], cancer vaccines [72], natural compounds [73,74]; (ii) molecular discovery—the comprehension of melanoma metastatic pathway involving microvascular environment [75]; and (iii) in vivo tracing of melanocytic lineage cells [76].

**Figure 1:**
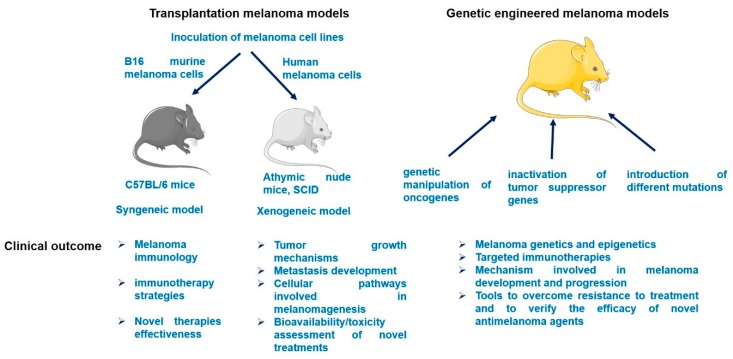
A schematic overview of murine melanoma models and their clinical outcomes.

Due to the considerable number of published melanoma models, in this review only the murine melanoma models will be briefly discussed: xenograft mouse models, genetic engineered models (GEM), and UV-induced models (Figure 1).

### 4.1. Xenograft Models

Mouse models applied for melanoma study have a long history [77] and exhibit several advantages compared to other animal models (fish, opossum, horse, dogs, pigs), such as: relevant known data concerning the genetic background which allows the possibility of genetic manipulation, easy breeding and handling, multiple studies about molecular pathways dissection, and representing appropriate hosts for patient-derived xenografts (PDXs) to develop semblable human disease and to establish personalized antimelanoma therapies [21,78].

The xenograft melanoma mouse models were obtained by inoculation of different melanoma cell lines into immunocompromised mice (summarized in Table 1) [50,79].

The xenograft models generated in immunocompromised mice by injection of human melanoma cells inoculum subcutaneously, exhibit a pathology semblable to human disease (after inoculation melanocytes proliferate and metastasize via lymphatic vessels and blood) and are frequently used to acquire data concerning the tumor growth mechanisms, the main cellular pathways involved in tumorigenesis, the bioavailability/toxicity associated to novel treatments [50].

This type of models proved to be successful in imitating the advanced metastatic melanoma, but was also employed to critically assess the behavior of melanoma cells *per se*, in terms of invasiveness, metastatic potential, and the role of stem cells [79]. The first immunocompromised mouse model built was an athymic nude/nude mouse in 1969, which allowed the growth of solid human tumors [80], followed by the CB17-SCID mice (which possessed natural killer (NK) cells and supported the xenograft of the human cells, but the tumor growth was limited) and NK-deficient NOD (Non-Obese Diabetic)/SCID (Severe Combined Immunodeficient) mice (which accepted the growth of most of the melanoma cells inoculated) [79].

In one of our recent studies, was proved that Balb/c athymic mice represent an eligible host for A375 achromic/amelanotic human melanoma cells, the primary tumors became well-defined at day 20 post-inoculation. Furthermore, lung metastases were detected at day 30 post-inoculation while by monitoring the survival time, the longer the survival, the lower the number of metastasis was recorded (an increased number of mast cells around the tumor was notified) [81]. As presented in the case of syngeneic models, there were some limitations observed for these models, like: the cultured melanoma cells lines are purified and show some differences compared to parent cells; during culture the cells might lose some metastatic promoting markers and the clinical outcome might be irrelevant [50].

### 4.2. Syngeneic Models

Syngeneic mouse models were obtained by inoculation of melanoma cells into mice that present the same genetic background (examples resumed in Table 1) [43]. The mice used for syngeneic allograft models are immunocompetent and these models are preferred to gather insights into the melanoma microenvironment by allowing inherent interactions between melanocytes and the immune cells [50].

One of the first syngeneic melanoma models was realized by Fidler and Kripke [77], which generated individual sublines of B16 mouse melanoma, the suspension of cells being thereafter administered intravenously to syngeneic C57BL/6 mice to verify their ability to form secondary tumors in lungs [38]. The B16 sublines remained the most common used cell lines for syngeneic transplantation, two of them, B16F1 (low metastatic potential—used for primary tumors development) and B16F10 (high metastatic potential—lung metastases) being well-established sub-clones and reliable platforms of data about melanoma immunology and immunotherapy strategies [50].

Another B16 subline described as a useful tool for in vivo melanoma models is B16 melanoma 4A5, a line derived from a cutaneous melanoma aroused in C57BL/6 mice that presents fibroblastic-like shape and ability to produce melanin, a feature that ceases after many passages in vitro [82]. Inoculation of B16 melanoma 4A5 cells intraperitoneally to C57BL/6 mice led to the development of lung and spleen metastases in less than 30 days post-administration [83], whereas subcutaneously injection of cells into C57BL/6 mice offers the possibility to assess the evolution of primary tumors and to test the effectiveness of different antimelanoma agents, the survival time being longer than in the case of intravenous or intraperitoneally inoculum [73,84]. The drawbacks of the syngeneic models consist of: (i) a short time frame between the appearance of the primary tumors and the metastasis occurrence, which impairs the pursuit of potential antimelanoma agents efficiency; and (ii) the murine origin of the cells that makes difficult the translation of the data obtained and might become inconsequent for human pathology [50].

### 4.3. Patient-Derived Tumor Xenografts (PDXs)

Another preclinical model of melanoma is represented by the patient-derived tumor xenografts model. This type of model consists of fresh tumor grafts collected from patients (under ethical approval) implantation into immunodeficient mice (athymic nude or NSG (NOD/SCID γ) mice) [20,50,80]. The use of human biopsies as xenografts offers several benefits as compared to cell lines, including: preservation of the clinical characteristics of parent tumor in terms of histology, transcriptome, polymorphism, DNA expression and sequence, and chromosomal architecture [20,80]. The heterogeneity of PDXs models and their ability to resemble the initial tumor made them suitable tools for the characterization of metastatic melanoma behavior, drug discovery, clinical response studies, identification of drug resistance and combined therapy effects, guidance in clinical management of melanoma patients, target identification, and validation strategies [20,67,71,80,92]. Besides the notable progress in melanoma preclinical and translational research field offered by PDXs models, there were also some drawbacks stated, such as: it is a time-consuming process that requires technical skills, a long-term experiment (three to nine months for melanoma development without a 100% rate of success), the lack of a fully functional immune system (the use of immunocompromised mice), inconveniencies for genetic manipulation, and high costs [20,50,80].

### 4.4. Genetically Engineered Models (GEM)

It is well-known that the transformation of a normal cell into a cancerous one involves a series of genetic and epigenetic changes, changes that were also described in melanoma [79]. In order to have a clear picture and to understand the role of these changes, many efforts were channeled to the development of genetic engineered melanoma mouse models. The genetic engineered models (GEMs) were performed using different approaches, such as: genetic manipulation of the ectopic expression of oncogenes, inactivation of tumor suppressor genes, and introduction of different mutations [39,79]. These models contributed significantly to depict the cells of origin by lineage tracing approaches (consists in labeling a single cell with a marker that offers visibility into the mechanisms involved in tumor initiation and progression of pre-neoplastic lesions) and to observe the results of the treatment administered, to identify the genes responsible for melanoma progression, and the molecular mechanisms associated with melanoma late stages [39,79,93]. In addition, GEMs proved to be reliable and reproducible models (present the same basic mutations) for evaluating the role of impaired genes/pathways and of the immune system cells in melanoma biology and treatment resistance. Other important features of GEMs consist of: the capacity to develop spontaneous melanoma tumors at their inherent site (spontaneous melanoma rarely occurs in adult mice), the ability to generate other mutations in order to verify their potential susceptibility or resistance to therapy, and the presence of a fully functional immune system that influences the tumor growth [39,94]. Besides the multiple applications of these models, there were also some limitations described: high costs and effort, the latency period for tumors appearance is long (9–12 months), and most frequently the mutagenic load is not similar with the one described in human tumors [20,50,94].

In order to obtain a relevant genetic engineered melanoma mouse model, there are some factors that should be considered, like: skin morphology (distinct anatomic features between human and murine skin), melanocytic lineage (the promoter used to drive expression to a specific cell type), carcinogenic agents (for the initiation or the enhancement of melanoma development), mice age (adult mice are more resistant at developing spontaneous melanoma), and skin microenvironment [94,95].

The first genetic engineered melanoma mouse model was the transgenic mouse model—Tyr-SV40, that exhibited overexpression of SV40 T antigen (Tag) under the control of melanocyte-specific tyrosinase (Tyr) gene promoter and developed melanoma spontaneously or after UV irradiation [2,94,95]. Linda Chin and her collaborators established the first mouse model—Tyr-HRAS by knocking out melanoma specific genes, *Cdkn2a*^−/−^, and developed spontaneous melanoma that do not metastasize [2,48,94]. The group of Dhomen built the *BRAF^V600E^* melanoma model that proved the necessity of additional genetic alterations to develop melanoma [94,96]. Subsequent studies showed that silencing *PTEN* gene in *BRAF^V600E^* melanoma model led to the development of metastatic melanoma [50,94,97]. Other genes that were manipulated to develop genetic mouse models of melanoma were: *RET*—a proto-oncogene that enciphers glial cell-derived neurotrophic factor-specific receptor tyrosine kinase and interferes in the progression stage of melanoma [50]; Mt1-hepatocyte growth factor/scatter factor—*Mt1-HGF/SF* [94,98]; G-protein-coupled receptor *GRM-1* (metabotropic glutamate receptor-1) [79]; guanine nucleotide-binding protein G(q) subunit α (*GNAQ*) [80]; and cyclin-dependent kinase 4 (*CDK4*) [2,20,95]. These basic genetic engineered models represented the starting point for more complex mouse models by induction of additional mutations and were clearly described in several studies [20,49,80,95].

Some excellent reviews of recent date have addressed the available genetic engineered mouse models of melanoma and their role in the advancement of human melanoma initiation and progression [20,21,95], thus this paper will offer a summarized version (see Table 2) of the described models obtained by overexpression of genes, in terms of: the gene mutation, animal strain, signaling pathways altered, and the promoter involved in melanoma development.

### 4.5. UV-Induced Mouse Melanoma Models

Ultraviolet radiation exposure is considered one of the main etiological factors involved in melanoma development, but until present a clear link between UV-induced DNA damage and melanoma initiation has not been established. Recent studies suggested that DNA impairment requires the presence of other dysregulated genes (via epigenetic events) to initiate UV-induced melanomagenesis [39].

To elucidate the UV radiation role in melanoma development, several animal models were proposed but some important setbacks were encountered, including: the localization of melanocytes in human skin (at epidermal–dermal junctions and within hair follicles) versus mice skin (most melanocytes are located in hair follicles, only few are found in epidermis, mainly in non-hairy skin—ears, footpad) [2,58,79], the diversity of human melanoma genetic, histopathological, and clinical features (numerous genes and signaling pathways) which hinders the development of a model that mirrors the characteristics of human melanoma [79].

The mice characteristics regarding the epidermal melanocytes (their number augments in the first 2 weeks after birth and starts to decline during hair follicles formation process when they cross the basement membrane) were thought to be responsible for the inability to develop spontaneous melanoma in adult mice after UV exposure (acute—intense, short-term or chronic—low doses) [2]. Still, there were developed melanoma models using hairless mice subjected to DMBA (7,12-dimethylbenzanthracene)—as initiator agent and UV irradiation promoter, and transgenic mice carrying *BRAF* mutations (increased risk to develop melanoma when UV irradiation was added) [33].

There were also recorded differences regarding melanocytes location in murine skin dependent on age: in newborn mice are located at the epidermal–dermal junction, whereas in adolescent and adult mice, melanocytes are restricted to hair follicles in corporal skin. Melanocytes and hair pigmentation are two connected processes since melanocytes play not only a pigmentary role, but also a hair growth-regulatory one being strictly coupled to the anagen phase of hair cycle [108,109].

The most successful protocols to develop melanoma in mice after UV exposure consisted of: exposure of genetic engineered mice overexpressing SV40 Tag and HGF/SF starting with day 4 after birth; these data being somehow in accordance with the results of multiple human epidemiological studies which assert that an increased exposure to sun in the childhood augments the risk for melanoma [2]. 

A relevant model of melanoma was considered to be the one initiated in neonatal transgenic for hepatocyte growth factor/scatter factor (HGF/SF) mice (after a single neonatal dose of mild erythemal UVB radiation), that presents melanocytes localized in the epidermis and the tumor arose in different phases similar with the ones described for human melanoma: benign nevus, radial growth phase, vertical growth phase, and late metastatic spread to other organs [58,80]. The mouse melanoma model obtained by UV irradiation is considered to be the most reliable model to clinically characterize melanomagenesis [80]. Other approaches to initiate melanomagenesis involved the application of DMBA, a polycyclic aromatic hydrocarbon with immunosuppressive effects and TPA (13-*O*-tetradecanoyl phobol acetate), a phorbol ester responsible for skin irritation and black lesions (that will transform to melanoma) after topical administration [88].

Our research group developed a photo-chemically induced skin carcinogenesis model using SKH-1 hairless mice and the association of UV irradiation and topical application of DMBA and TPA solutions, and the tumors resulted were non-melanoma skin cancer type with a high resemblance to human pathology (the incidence was higher in male mice) [110]. Even though the chemically induced melanoma models are frequently used for the evaluation of immune therapies effects on tumor growth, one of the main drawbacks is represented by the fact that the cells arising from the lesions induced are nonpigmented and do not recreate precisely the human pathology [80].

## 5. Conclusions

The impressive advances in melanoma biology, immunology, genetics, and epigenetics comprehension represent palpable reasons for an optimistic future for targeted treatments and immunotherapy. The information provided by mouse models managed to fill some of the gaps existent in melanoma knowledge and offer an arsenal to prevail the new challenges and setbacks associated to heterogeneity of melanoma; still, further considerable efforts are required to conceal melanoma complexity. Nonetheless, melanoma remains a very aggressive type of cancer with a high mortality rate, particularly in advanced stages, and a central topic for prospective cancer research. 

## Figures and Tables

**Table 1:** Several examples of xenotransplanted (xenogeneic and syngeneic) melanoma mouse models.

Model Type	Cell Line Inoculated	Strain of Mice	Type of Melanoma Developed	Clinical Outcome	References
Xenogeneic	MV3—melanoma cell line derived from transplanted fragments of a fresh human melanoma metastasis subcutaneously (s.c.) into a nude mouse	Nude mice	Metastatic melanoma in lungs	Useful tool to evaluate the antimetastatic potential of different agents	[85]
Xenogeneic	A375—human melanoma cell line	Balb/c nude mice	Cutaneous melanoma	To assess the metastatic potential of the cells in vivo and as further tool for testing novel melanoma agents	[81]
Xenogeneic	A375 human melanoma cells (s.c. inoculum)	NOD/SCID mice	Cutaneous melanoma	To test the efficiency of the oncolytic virus VSV-GP against metastatic melanoma	[86]
Xenogeneic	518A2 melanoma cells with *BRAF V600E* mutation and *CDKN2A* exon 2 deletion (s.c. inoculum)	Athymic nude mice (Harlan Winkelmann, Germany)	Cutaneous melanoma	To elucidate the mechanism of action of gliotoxin, an inhibitor of canonical NOTCH2/CSL transactivation (a signaling pathway detected in multiple human neoplasms)	[70]
Xenogeneic	A375 human melanoma cells (s.c. inoculum)	Athymic nude mice	Cutaneous melanoma	To evaluate itraconazole as possible inhibitor in melanoma and to establish its mechanism of action	[87]
Xenogeneic	UACC 903 and 1205 Lu melanoma cells	Athymic-Foxn1^nu^ nude mice	Melanoma	To verify the efficacy/toxicity of a combination of drugs (Celecoxib and Plumbagin) formulated as nano-delivery system against melanoma	[88]
Syngeneic	Harding-Passey melanoma cells	Balb/c and DBA/2F1 mice	Intracranial tumors	To study or modulate immune responses—Th2 response	[79,80]
Syngeneic	Cloudman S91 melanoma	DBA/2 mice	Melanoma	To evaluate the effectiveness of novel anticancer therapies and drug delivery platforms	[79,80]
Syngeneic	B16 melanoma cell lines	C57BL/6 mice	Melanoma	To produce tumor line variants with organs preferences and to test the efficacy of immunotherapy (cytokines, vaccines)	[79,80,89,90]
Syngeneic	B164A5 melanoma cell line (s.c. inoculated)	C57BL/6J mice	Melanoma	To assess the antimelanoma effects of betulinic acid, a natural compound	[73]
Syngeneic	B164A5 melanoma cell line intraperitoneally (i.p. inoculated)	C57BL/6	Metastatic melanoma	To gather data regarding tumor progression and metastasis	[83]
Syngeneic	B16-OVA melanoma cells (s.c. inoculum)	C57BL/6J	Cutaneous melanoma	To test the efficiency of the oncolytic virus VSV-GP against melanoma	[86]
Syngeneic	B16-OVA melanoma cells intravenously (i.v. injection of cells suspension)	C57BL/6J	Lung metastatic melanoma	To test the efficiency of the oncolytic virus VSV-GP against metastatic melanoma	[86]
Syngeneic	B164A5, B16F10, B16GMCSF, B16FLT3 melanoma cells	C57Bl/6J	Metastatic melanoma	To verify the metastatic potential of the cells	[84]
Syngeneic	B16.OVA melanoma cells (s.c. inoculum)	C57BL/6J	Cutaneous melanoma	To check the antitumor potential of dasatinib, a specific BCR/ABL and SRC-family tyrosine kinase inhibitor	[91]

**Table 2:** Representative examples of genetic engineered mouse (GEM) models of melanoma.

GEM Name	Gene Mutation	Animal Strain/Background	Signaling Pathways Altered	Promoter ± Carcinogen	References
Tyr-SV40 Tag (high expression)	SV40 T antigen (Tag)-overexpression	C57/BL6	pRB (p16)/p53 (ARF)	Tyr	[2,80,99]
Tyr-SV40 Tag (low expression)	SV40 T antigen (Tag)-overexpression	C57/BL6	pRB (p16)/p53 (ARF)	Tyr + UV radiation	
(MT1)-Ret TRP1-Ret (G12V)	Ret proto-oncogen-overexpression	NMRI C3H	MAPK (Ras)/MAPK (Raf)/PTEN/Akt Ras and PI3K	Mt1 + UV radiation	[2,100,101]
Mt1-HGF/SF	HGF/SF-overexpression	FVB	MAPK (p38^MAPK^) MAPK (Ras)/MAPK (Raf)/PTEN/Akt	Mt1 + UV radiation	[2,20,98,102,103]
Krt4-Scf	Scf-overexpression	C57/BL	Kit receptor, MAPK	Krt4	[2,104]
Tyr-Hras (G12V)	Hras (G12V)-oncogene overexpression	Mixed	MAPK (Ras)/MAPK (Raf)/PTEN/Akt	Tyr + DMBA or UV	[105]
Tyr::NRAS^Q61K^	NRAS (NRAS^Q61K^)-overexpression	Tyr::N-Ras^Q61K^ transgenic mice	pRB (p16)/p53 (ARF)/MAPK (Ras)/MAPK (Raf)/PTEN/Akt	Tyr	[20,106]
Hgf-Cdk4^R24C^	Overexpression of HGF and an oncogenic mutation CDK4^R24C^	HGF × CDK4^R24C^ C57BL/6 mice	pRB (p16)	MT1+ DMBA and TPA	[20,107]
Braf^CA^	Braf^V600E^ from the endogenous Braf gene	Tyr::CreER; BRaf^CA/+^; Pten^lox5/lox5^ mouse	BRAF→MEK1/2→ERK1/2 MAPK PTEN→INK4A and/or ARF	Tyr::CreER^T2^ + UVB	[97]
Hairless RFP–RET-transgenic mice of line 304-hr/hr-HL-RET mice		Crossings of RET-mice with HL-mice (Hos:HRM) under C57BL/6J background	MAPK (Ras)/MAPK (Raf)/PTEN/Akt		[65]

## Abbreviations

A375	Human melanoma cells
ACTH	Adrenocorticotropic hormone
AJCC	American Joint Committee on Cancer
B16.OVA	Murine melanoma cell line
B164A5	Murine melanoma cell line
B16F10	Murine metastatic melanoma cells
B16FLT3	Murine melanoma cell line
B16GMCSF	Murine melanoma cell line
BRAF	Human gene that encodes B-Raf protein
CDK4	Cyclin-dependent kinase 4
CDKN2A	Cyclin-dependent kinase N2A
DMBA	7,12-dimethylbenzanthracene
DNA	Deoxyribonucleic acid
EMT	Epithelial-to-mesenchymal transition
ERK	Extracellular signal-regulated kinase
EUROCARE 5	European Cancer Registry based Study on Survival and Care of Cancer Patients
GEM	Genetically engineered models
GLOBOCAN	Global Cancer Incidence, Mortality and Prevalence
GNAQ	Guanine nucleotide-binding protein G(q) subunit α
GPCR	G-protein-coupled receptor
GRM-1	Metabotropic glutamate receptor-1
HGF/SF	Hepatocyte growth factor/scatter factor
i.p.	Intraperitoneally
i.v.	Intravenously
IAPs	Inhibitory apoptosis proteins
l-DOPA	l-dihydroxyphenylalanine
MAPK	Mitogen-activated protein kinase
Mb	Megabase
MC1 receptor	Melanocortin 1 receptor
Mt1	Metallothionein
Mt1-HGF/SF	Mt1-hepatocyte growth factor/scatter factor
NF1	Neurofibromin 1
NK	Natural killer cells
NRAS	Neuroblastoma RAS viral oncogene homolog
p14-ARF	Tumor suppressor protein
p16-INK4A	Tumor suppressor protein
PDXs	Patient-derived tumor xenografts
PI3K	Phosphatidylinositol-3-kinase pathway
PTEN	Phosphatase and tensin homolog protein
RGP	Radial growth phase
s.c.	Subcutaneously
SLUG	Transcriptional repressor of E-cadherin
SNAIL	Transcription factor
TCGA	The Cancer Genome Atlas
TP53	Tumor protein p53
TPA	13-*O*-tetradecanoyl phobol acetate
TWIST	Twist-related protein 1
Tyr	Melanocyte-specific tyrosinase gene promoter
UV	Ultraviolet
WHO	World Health Organization
ZEB1	Zinc finger E-box binding homeobox 1 transcription factor
